# Aligning Dietary Intake and Food Purchase Data in United States Older Adults: Insights from the National Health and Nutrition Examination Survey and Circana Consumer Network

**DOI:** 10.1016/j.cdnut.2026.109421

**Published:** 2026-06-29

**Authors:** Naglaa H El-Abbadi, Joshua Rutt, Marcus Lehr, Frederick Nusetor, Cristina Moraga Franco, Andrea C Carlson, M. Kyla Shea, Chao-Qiang Lai, Laurence D Parnell, Eric Miller, Jose M Ordovas

**Affiliations:** 1 Jean Mayer USDA Human Nutrition Research Center on Aging, at Tufts University, Boston, MA, United States; 2 Retired From Economic Research Service, U.S. Department of Agriculture, Washington, DC, United States; 3 Friedman School of Nutrition Science and Policy, Tufts University, Boston, MA, United States; 4 USDA ARS, Jean Mayer USDA Human Nutrition Research Center on Aging at Tufts University, Boston, MA, United States; 5 Department of Electrical and Computer Engineering, Tufts University, Boston, MA, United States

**Keywords:** nutrition datasets, dietary intake, food purchase, diet quality, older adults, NHANES, Circana Consumer Network, Purchase-to-Plate Crosswalk (PPC), Healthy Eating Index (HEI)

## Abstract

**Background:**

Diet quality is a key determinant of healthy aging, yet fully assessing dietary intake remains a challenge partly because of data limitations and difficulty in integrating information from methodologically distinct datasets. Different nationally representative datasets can offer complementary perspectives: the National Health and Nutrition Examination Survey (NHANES) captures self-reported dietary intake using 1 to 2 d of 24-h recall, whereas the Circana Consumer Network (CN) is a national retail dataset that records year-long grocery purchases from ∼60,000 United States households.

**Objectives:**

This study aimed to evaluate whether dietary intake reported in NHANES 2017–2018 aligns with food purchase trends in CN 2018 among United States adults aged ≥55 y.

**Methods:**

We examined self-reported dietary intake of grocery/store-bought foods from 24-h recalls in NHANES (*n* = 1950 individuals) and, separately, household grocery purchases from 1- and 2-person households in CN (*n* = 29,192 households), analyzed by sex and age group (55–64 y, 65–74 y, and 75+ y). For each dataset, healthfulness of food intakes or purchases was assessed using the 2015 Healthy Eating Index (HEI) and food group proportions were evaluated by percent mass (grams) and energy (kilocalorie).

**Results:**

Mean total HEI scores were modest and comparable across datasets (NHANES: 55.7 ± 0.9; CN: 51.3 ± 0.1; *P* = 0.004). Both sources demonstrated moderate agreement among the 13 HEI component scores, revealing consistent shortfalls in total/whole fruit, greens and beans, whole grains, and saturated fat moderation. Food group proportions had <10% difference across most categories by both mass and energy, although there were higher discrepancies for mixed dishes, condiments, and snacks/sweets.

**Conclusions:**

In United States adults aged ≥55 y, grocery/store-sourced intake from NHANES and household grocery purchases from CN showed broadly similar but not interchangeable patterns of diet quality, indicating that purchase data can complement, but not replace, recall-based dietary surveillance. Integrating purchase records with recalls could provide deeper insights into older adults’ dietary behaviors and strengthen precision nutrition research.

## Introduction

Older adults in the United States spend an estimated 10 years at the end of life in poor health, due in large part to diet-related diseases that contribute significantly to the burden of disability [[1], [2], [3]]. This discrepancy between healthspan and lifespan is associated with substantial personal, societal, and economic costs [2]. Improving dietary quality has the potential to prevent or mitigate many age-related conditions, yet national data suggest that older adults fall short of meeting dietary recommendations [1,3].

Accurate dietary assessment is essential to evaluate and improve diet quality across the lifespan and shape effective recommendations. However, dietary intake across the full span of older age remains poorly characterized, and with little differentiation across population groups [4]. The NHANES is the most comprehensive tool for dietary surveillance in the United States, providing self-reported intake data through 24-h recalls alongside clinical and demographic measures [5], making it a rich source of dietary data and trends [6]. Yet, although robust, these data are subject to recall bias, underreporting, and logistical limitations that constrain their frequency and scope [7,8].

Consumer food purchase datasets offer an alternative lens into food behaviors—by capturing item-level purchases over long timeframes [[9], [10], [11]]. The Circana Consumer Network (CN) is a large, demographically representative panel that tracks year-long grocery purchases from over 60,000 households in the United States using barcode scanning. One significant limitation, however, is that these databases typically do not include the foods’ nutritional content details beyond the nutrition facts label information for universal product codes (UPCs), making it difficult to compare diet patterns across datasets because of the lack of dietary intake data or detailed dietary information for food purchases. When linked with nutrient databases via the USDA’s purchase-to-plate crosswalk (PPC), these data can be used to evaluate the healthfulness of household food purchases [12].

The individual methodological strengths of these datasets may be combined for a more comprehensive nutritional assessment. Some previous studies have examined how well self-reported dietary intake aligns with food purchase data [[13], [14], [15]], but none in the United States at the national level, particularly among older adults. This is a critical gap, given the aging of the United States population and the potential to enhance dietary surveillance by triangulating multiple data sources.

Therefore, our objective was to compare the healthfulness of grocery/store-sourced foods among United States adults aged ≥55 y in NHANES 2017–2018 and CN 2018, by assessing concordance in diet quality [Healthy Eating Index (HEI)-2015] and food group proportion by mass and energy. We hypothesized that total HEI scores and food group proportions would be broadly similar across datasets, but discrepancies would show how 2 methodologically distinct datasets can complement each other by providing different/nonoverlapping information.

## Methods

### Study design and data sources

We conducted a cross-sectional analysis of dietary intake and household food purchase behaviors among United States adults aged ≥55 y using 2 nationally representative datasets: the 2017–2018 cycle of the NHANES and the 2018 National Consumer Panel (NCP), distributed by Circana, as the CN. NHANES uses a complex, multistage sample design, with weight adjustment to represent the noninstitutionalized population residing in the United States [16]. Self-reported dietary data of individual participants are obtained through structured 24-h recalls administered by trained interviewers [5]. Meanwhile, the NCP is a joint venture between the market research companies, Circana and NielsenIQ, and captures household food-at-home purchases from a large panel of United States households using barcode scanning. These households are selected for participation using quota-based sampling meant to demographically represent the population in the United States census [9]. Household shopping trips record item-level food purchases and associated information. Additionally, each household has accompanying demographic information, some of which is recorded for individual members of the household (e.g., age, sex, and educational attainment) and some of which is recorded at the household level (e.g., income, household size, presence of children, residential region, etc.) [9]. CN data were linked to USDA nutrition databases via the PPC [17], enabling comparability with dietary intake data.

### Sample selection

**FIGURE 1:**
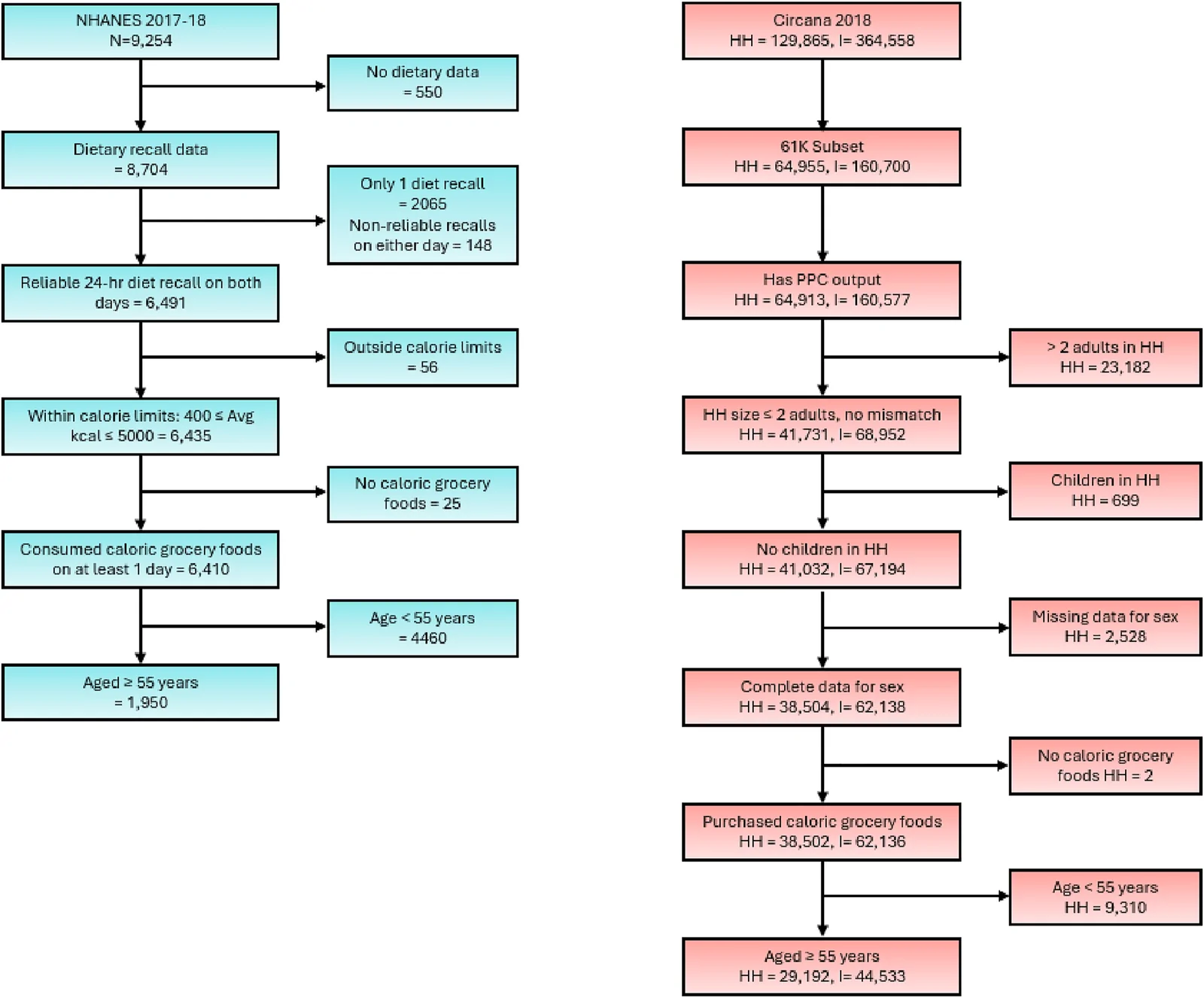
Final analytic samples for NHANES and Circana (CN).

For NHANES, participants were included in the analysis if they were aged 55 y or older, completed 2 reliable 24-h dietary recalls, reported an average daily caloric intake between 400 and 5000 kcal, and consumed >0 kcal from food sourced at grocery or other stores on ≥1 recall day. The source of each food item was identified using NHANES variables DR1FS and DR2FS, and only items coded as grocery/store-bought were retained, which represented ∼70% of the foods. The remaining 30% of food items obtained from nonstore sources (e.g. restaurants) were excluded. This yielded a final analytic sample of 1950 individuals from NHANES (Figure 1).

The full CN dataset was of households participating in year-long reporting of grocery purchases using handheld scanners or mobile applications, and those meeting the minimum criteria of expenditures (minimum average spending amount of $25 or $35 per week for 1- or 2-person households, respectively) and scanning frequency (reporting purchases at least once every 4 wk for 80% of the time periods, or for 11 of the 13 four-week reporting periods for the year) were included as part of the static sample dataset [18]. The CN sample analyzed here was restricted to 1- or 2-person households in the static sample, with complete demographic information for both age and sex, and no presence of children. Only members aged 55 y or older were included in the sample. All purchases were identified at the UPC level and aggregated at the household level. The final analytic sample included 29,192 households, representing 44,533 individuals (Figure 1, Supplemental Figure 1).

In addition to age and sex, demographic characteristics for both datasets were also assessed for race/ethnicity, marital status, education level, and family household poverty income ratio (PIR), assessed in relation to the Federal poverty threshold. The categories were aligned across the 2 datasets. For PIR, category thresholds were selected at 1.3, 1.85, and 4, as these corresponded with eligibility for the Supplemental Nutrition Assistance Program and the Special Supplemental Nutrition Program for Women, Infants, and Children, and these cutoffs were in line with differentiating middle- and high-income families for the range of incomes in these 2 datasets [19].

### Dietary and purchase measures

In NHANES, dietary intake was captured using the USDA Automated Multiple-Pass Method for 24-h dietary recall and linked to the Food and Nutrient Database for Dietary Studies (FNDDS) [20]. This analysis was restricted to grocery or retail store-sourced foods to ensure comparability with the CN dataset, which includes only store-based purchases. In CN, information on item-level food purchases was obtained from scanner data entered continuously for participant shopping trips throughout the calendar year of 2018. Approximately 550,000 food and beverage products with sales data were individually mapped to nutritional and food group data via the PPC developed by USDA researchers at the Economic Research Service [12,21]. The PPC translated the purchased forms of food items in CN to analogous forms in the FNDDS, by determining conversion factors to adjust the weight of food items at the UPC level so as to match the corresponding FNDDS food through removal of inedible weight (i.e., from rinds, seeds, bones, etc.) [17]. This allowed for detailed classification of food group composition by percentage weight (grams) and percentage of energy (kilocalories).

### Diet quality assessment (HEI-2015) and diet composition

Healthfulness of grocery/store-bought foods in both datasets was evaluated using the HEI-2015, which scores diet quality based on adherence to the Dietary Guidelines for Americans [23]. As shown in Table 1, the HEI includes 13 components encompassing both adequacy (e.g., whole fruit, total vegetables, and whole grains) and moderation (e.g., sodium, saturated fat, and added sugars) components [23,22]. HEI scores are calculated per 1000 kcal, and component scores are scaled proportionally based on established thresholds from the Dietary Guidelines [24,25]. HEI scores were previously determined by Economic Research Service (ERS) researchers for the ∼550,000 products covered by the 2017–2018 PPC. This was done by calculating the total weight of the converted forms of all food items from each household’s year of purchases and proceeding with HEI scoring calculations per 1000 kcal of foods purchased [12].

**TABLE 1:** Healthy Eating Index 2015 scoring principles^1^

Component	Maximum points	Standard for maximum score	Standard for minimum score of 0
Adequacy components			
Total fruits^2^	5	≥0.8 cup eq/1000 kcal	No fruit
Whole fruits^3^	5	≥0.4 cup eq/1000 kcal	No whole fruit
Total vegetables^4^	5	≥1.1 cup eq/1000 kcal	No vegetables
Greens and beans^4^	5	≥0.2 cup eq/1000 kcal	No dark green vegetables or legumes
Whole grains	10	≥1.5 oz eq/1000 kcal	No whole grains
Dairy^5^	10	≥1.3 cup eq/1000 kcal	No dairy
Total protein foods^4^	5	≥2.5 oz eq/1000 kcal	No protein foods
Seafood and plant proteins^6^	5	≥0.8 oz eq/1000 kcal	No seafood/plant proteins
Fatty acids^7^	10	(PUFAs + MUFAs)/SFAs ≥2.5	(PUFAs + MUFAs)/SFAs ≤1.2
Moderation components			
Refined grains	10	≤1.8 oz eq/1000 kcal	≥4.3 oz eq/1000 kcal
Sodium	10	≤1.1 g/1000 kcal	≥2.0 g/1000 kcal
Added sugars	10	≤6.5% of energy	≥26% of energy
Saturated fats	10	≤8% of energy	≥16% of energy

This table is recreated with some modifications from Krebs-Smith et al. 2018 and Shams-White et al. 2023, which provide more detailed description of component coverage, allocation, and scoring methods [22,23].

1 The Healthy Eating Index-15 component intakes between the minimum and maximum standards are scored proportionately. Component scores are summed to create the total score.

2 Includes 100% fruit juice.

3 Includes all forms except juice.

4 Includes beans, peas, and lentils.

5 Includes all milk products, such as fluid milk, yogurt, and cheese, and fortified soy beverages.

6 Includes seafood, nuts, seeds, soy products (other than beverages), and beans, peas, and lentils.

7 Ratio of PUFAs and MUFAs to SFAs.

The simple means method was applied in both NHANES and CN data to ensure methodological consistency. Although the population ratio (PR) method better estimates habitual population-level intake from limited recalls, it was not applied to CN data, which already reflects long-term behavior. For NHANES, intake values of grocery/store-bought foods were summed across 2 recall days for each individual, whereas in CN, purchases were summed across the full year for each household. Additional assessment using the PR method was performed on the NHANES dataset, as PR is generally recommended for estimating habitual population-level HEI scores from limited recalls [26].

Food group proportions were computed for the 9 major food groups defined by the 2015 Dietary Guidelines Advisory Committee (DGAC) in reported intake (NHANES) and household purchases (CN): dairy; protein foods; mixed dishes; grains; vegetables; fruits and 100% fruit juice; beverages (not including milk); condiments, spreads, and gravies; snacks and sweets [27]. These were analyzed by the percentage of total weight (grams) and the percentage of total energy (kilocalories).

### Statistical analysis

All statistical analyses were conducted using SAS version 9.4 (SAS Institute Inc.) and R version 4.4.3 (R Core Team, 2025) using the survey package (v4.4.2; Lumley, 2024) to calculate weighted estimates. NHANES data were weighted using the 2-d dietary recall weights [16], with the strata and nested primary sampling unit (PSU) structure taken into account via the survey package in R. CN analyses were conducted within the secure Administrative Data Research Facility computing enclave. A single set of demographic sampling weights was applied with no strata or PSU information, as they are not used in this survey. Weighted estimates were generated from a mix of the R survey package and SAS’s PROC SURVEYMEANS and SURVEYFREQ procedures. In both datasets, subgroup analyses were performed using a domain variable to ensure that all weights were considered for accurate variance estimation. Comparisons between datasets were of group means at the population group level and were evaluated using independent samples t tests with unpooled variances (Welch’s t test). Single dataset comparisons were done using Rao–Scott corrected χ^2^ tests for categorical variables.

Subgroup analysis by age and sex was conducted in both CN and NHANES. Because HEI and food group composition values were derived at the household level in the CN dataset, some level of disaggregation was required for subgroup analyses, requiring duplicate observations per household in 2-person households. For age-based analyses, individuals from 2-person households in CN were assigned to their respective age groups (55–64 y, 65–74 y, and 75+ y). For sex-based analyses, the male subgroup in CN included male-only households (either 1- or 2-person), and the female subgroup included female-only households (Supplemental Figure 1). A separate category was created for mixed-sex (male/female) 2-person households in CN, although this group was not used for direct comparisons with NHANES.

## Results

### Population characteristics

Table 2 presents the weighted demographic characteristics of the NHANES (*n* = 1950) and CN (*n* = 44,533 households) samples, stratified by age groups of 55 to 64 y, 65 to 74 y, and 75 y and above (50%, 32%, and 18% in NHANES, and 38%, 35%, and 26% in CN, respectively). In both datasets, females were more represented than males across all age categories, particularly in the oldest group (≥75 y). The majority of households were non-Hispanic White, based on the self-reported race/ethnicity of the primary respondent, with this proportion increasing with age in both datasets; all other race/ethnic groups had higher proportions in the 55 to 64 y category than the 2 older age groups. Educational attainment of the primary respondent, as well as household poverty income ratios, showed broad alignment between the 2 datasets. Most participants in NHANES and CN were married or living with a partner, but the eldest group in both datasets had ∼40% who were widowed, divorced, or otherwise separated. A minority were single or never married, but these proportions were statistically significant within each age group across the 2 datasets (*P* < 0.01) (Table 2). Otherwise, household marital status in the CN was similar to that in NHANES. There were also differences among the proportion of education level for “<12th grade” (higher in NHANES) and “High school or some college” (higher in Circana) for the middle and older age groups (*P* < 0.01). Overall, however, demographic alignments support comparability in dietary analyses across both data sources.

**TABLE 2:** Population characteristics (weighted proportions) in NHANES and CN datasets, by age group (NHANES unweighted *n* = 1950; CN unweighted *n* = 29,192; 44,533)

Characteristics	Age 55–64 y			Age 65–74 y			Age ≥75 y		
	NHANES (%)	CN (%)	*P* value	NHANES (%)	CN (%)	*P* value	NHANES (%)	CN (%)	*P* value
*n*	50	38	<0.001	32	35	0.25	18	26	<0.001
Sex									
Female	54	58	0.39	53	56	0.99	57	60	0.92
Male	46	42		47	44		43	40	
Race/ethnicity									
Non-Hispanic White	67	76	0.31	79	80	1.0	81	85	1.0
Non-Hispanic Black	10	12	1.0	7	10	1.0	7	8	1.0
Hispanic/Latino	12	7	0.08	8	6	1.0	6	4	0.77
Asian	6	3	0.13	3	2	1.0	4	2	0.43
Other/multiple	5	3	0.52	2	2	1.0	1	2	1.0
Marital status									
Married/living with partner	73	66	0.12	68	65	1.0	57	54	1.0
Widowed/divorced/separated	21	21	1.0	29	26	1.0	41	41	1.0
Never married/single	6	13	<0.001	3	9	<0.001	2	5	0.006
Education									
<12th grade	8	5	0.11	11	4	0.001	14	7	0.004
High school or some college	64	65	1.0	51	64	0.12	50	66	0.003
Bachelor's degree or above	28	30	1.0	39	31	1.0	36	28	0.46
Poverty income ratio									
0 to <1.3	16	13	1.0	9	13	0.48	14	14	1.0
1.3 to <1.85	10	10	1.0	9	13	1.0	14	16	1.0
1.85 to <4	32	27	1.0	34	33	1.0	40	40	1.0
≥4	42	50	0.64	48	41	1.0	32	30	1.0

Estimates are calculated using survey design weights. Comparisons of estimates between datasets were evaluated using independent samples t tests with unpooled SE estimates. Comparisons for multiple categories within each characteristic, and for 3 age groups, are adjusted using the Bonferroni method. For unweighted sample sizes: NHANES, *n* = 1950 adults aged ≥55 y; for CN, *n* = 29,192 households representing 44,533 adults aged ≥55 y.

Abbreviation: CN, Consumer Network.

### Total HEI scores by age and sex

**FIGURE 2:**
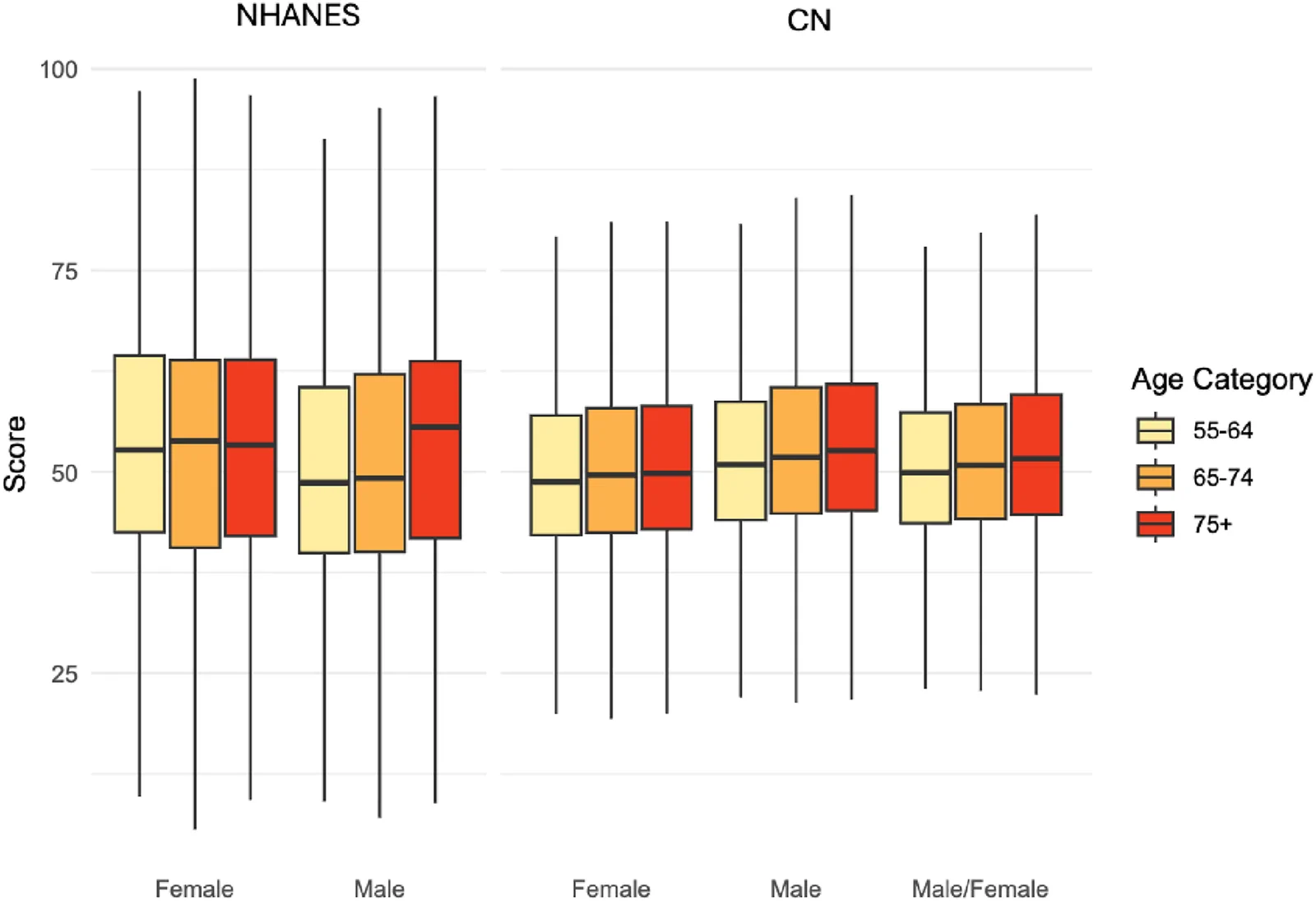
Total HEI by age group and sex in NHANES and Circana (CN). In CN, households (HHs) were differentiated as follows: female: 1- or 2-p HHs all female, male: 1- or 2-p HHs all male, male/female: 2-p HHs mixed male and female. HEI, Healthy Eating Index; p, person.

Figure 2 displays the total HEI-2015 scores of grocery/store-bought foods by age group and sex in both NHANES and CN. In NHANES, females in the different age categories showed similar median HEI scores, of ∼53, whereas males showed higher median HEI scores in the eldest group comparatively, from <50 in the 55 to 74 y groups to 55.6 in the ≥75 y group. In CN, median and lower/upper quartile values of total HEI scores were slightly higher in the older age groups, regardless of sex. The interquartile ranges were narrower in CN than in NHANES.

The mean total HEI score was 55.7 ± 0.9 in NHANES and 51.3 ± 0.06 in CN (*P* = 0.004), with neither dataset showing more than a 3-point difference in total score in either direction across age or sex (Supplemental Table 1). Between the 2 datasets, the mean total HEI in NHANES was slightly higher than in CN, by 1% to 7%, depending on the population group. This difference was consistently lower for males of all age groups (1%–4%) and higher for females, possibly indicating less alignment between grocery food intake and purchase for females (data not shown).

### HEI component scores by dataset and age group

**FIGURE 3:**
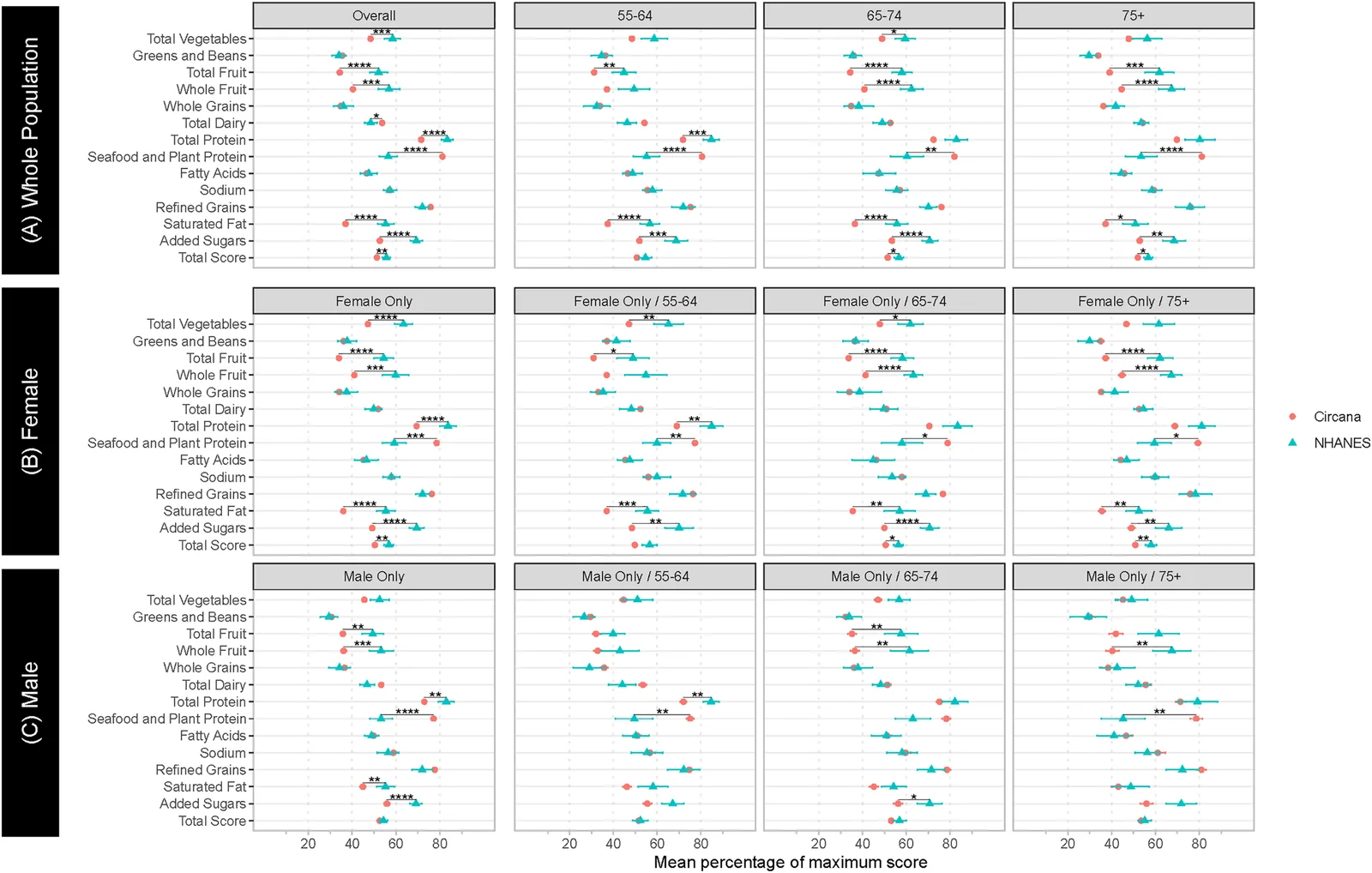
HEI component and total mean scores as percent of maximum points, with SE bars, displayed by dataset and age/sex subgroups. Black lines and stars indicate significance. ∗ = <0.05, ∗∗ = <0.01, ∗∗∗ = <0.001, and ∗∗∗∗ = <0.0001. (A) whole population, (B) female, (C) male. HEI, Healthy Eating Index.

Figure 3 presents HEI 2015 total and component scores in NHANES and CN in 3 rows of panels: (A) all participants of each dataset, as well as comparisons by age group (B), female, overall and by age group (C), male, overall and by age group. HEI components are scored out of 5 or 10 points; therefore, values were transformed into percent maximum to standardize across components for Figure 3 (with 100% indicating perfect adherence to dietary guidelines). Direct values may be viewed in Supplemental Table 1. In Figure 3(B) and (C), 2-person mixed-sex households from the CN dataset are not shown as they do not have a direct comparison in NHANES. See Supplemental Figure 1 for subgroup sample sizes.

There was moderate agreement in the 13 HEI component scores between the 2 datasets: 5 did not differ significantly between NHANES and CN in the full samples (*P* > 0.5), 1 component score differed by 5% (*P* < 0.05), and 7 differed by 10% to 25% (*P* < 0.0001). In both datasets, HEI component scores were consistently lowest (at or below around 40% of adherence guidelines) for greens and beans and whole grains, particularly among males; thresholds were better met by older age groups for whole grains, but not for greens and beans.

In CN purchases, there were also generally low levels (∼50% or less of adequacy thresholds) of total vegetables, total fruit, and whole fruit. Total dairy ranged from 50% to 56%. Fatty acids and saturated fats showed suboptimal performance across groups, in terms of adequacy for the ratio of MUFA and PUFAs to saturated fats, with 44% to 51% meeting guidelines across population groups, or 36% to 46% meeting moderation limits for percent energy from saturated fats, indicating that saturated fats are often present in household purchases at above recommended levels. Sodium and added sugars generally ranged from 50% to 60%. HEI components closest to the recommended guidelines in CN were refined grains, total protein, and seafood and plant protein, which were mostly 70% and above.

In reported grocery food consumption in NHANES, there was also a low intake of total dairy (44%–54%). Fatty acids and sodium were at similar levels as CN (48%–51% and 55%–60%, respectively), but saturated fats and added sugars performed better in NHANES than in CN, at 49% to 58% and 66% to 72% for moderate intake. Refined grains and total protein were also high at 70% or well above.

In NHANES, significant differences by age were observed for total and whole fruit, with the 55 to 64 y group scoring significantly lower than those aged 65 y and older (*P* < 0.05). Other components—including greens and beans, whole grains, total dairy, and saturated fat—showed some numerical variation but lacked statistical significance. In CN, variation across age groups was evident primarily for total and whole fruit purchases and sodium level. Compared with NHANES, CN showed higher consistency by age group across HEI components.

Notable cross-dataset discrepancies were found in scores for seafood and plant proteins, saturated fats, and added sugars, with significant differences between reported consumption and purchase data (*P* < 0.001 for overall, *P* < 0.05 across age groups). However, both datasets showed strong alignment (i.e., least difference in mean scores) in components such as whole grains, total dairy, refined grains, and sodium.

**FIGURE 4:**
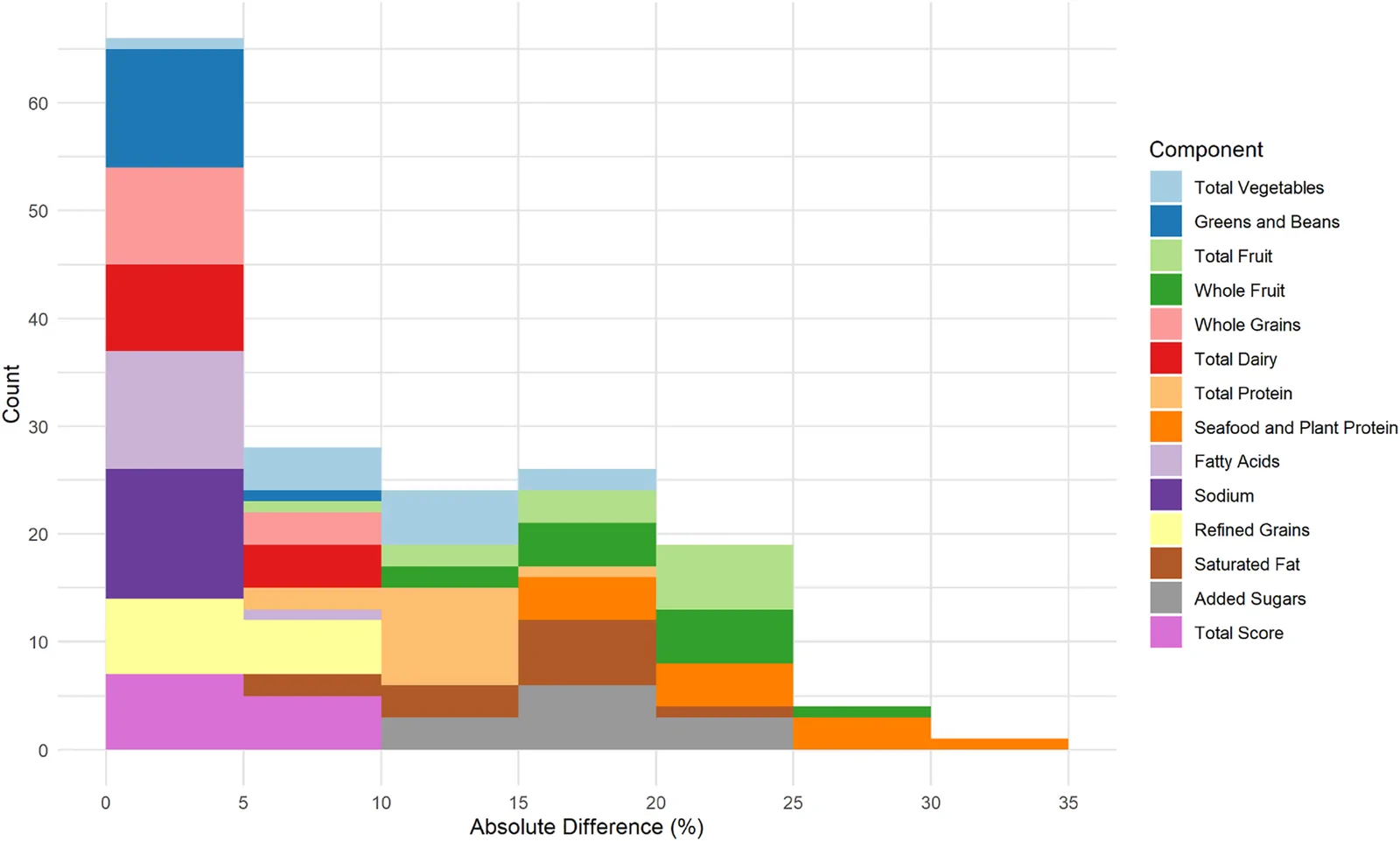
Absolute difference (%) between HEI total and component means in Circana (CN) compared with NHANES. For each data pair shown in Figure 3, the absolute difference is taken, and these values are binned and colorized by component. HEI, Healthy Eating Index.

Additionally, standardized differences between CN and NHANES are shown in Figure 4 for the 13 HEI component means as well as mean total HEI (all together 14 values), for the 12 population subgroups (overall as well as distinguished by age group and/or sex; e.g., ages 55–64 y for both males). This was calculated as standardized values of the absolute differences in means of the 13 HEI component scores and the mean total HEI the 12 population groupings (overall as well as distinguished by age and/or sex—note that some of these groupings were therefore inclusive) of both CN and NHANES (Figure 4). Of the 168 values, 66 showed <5% difference, and 26 were >20% different. The greatest discrepancies were in seafood and plant proteins (15%–35%), whole fruit (15%–30%), total fruit (5%–25%), added sugars (10%–25%), and saturated fats (5%–25%) (Figure 4).

### Comparison of HEI scoring methods in NHANES

**FIGURE 5:**
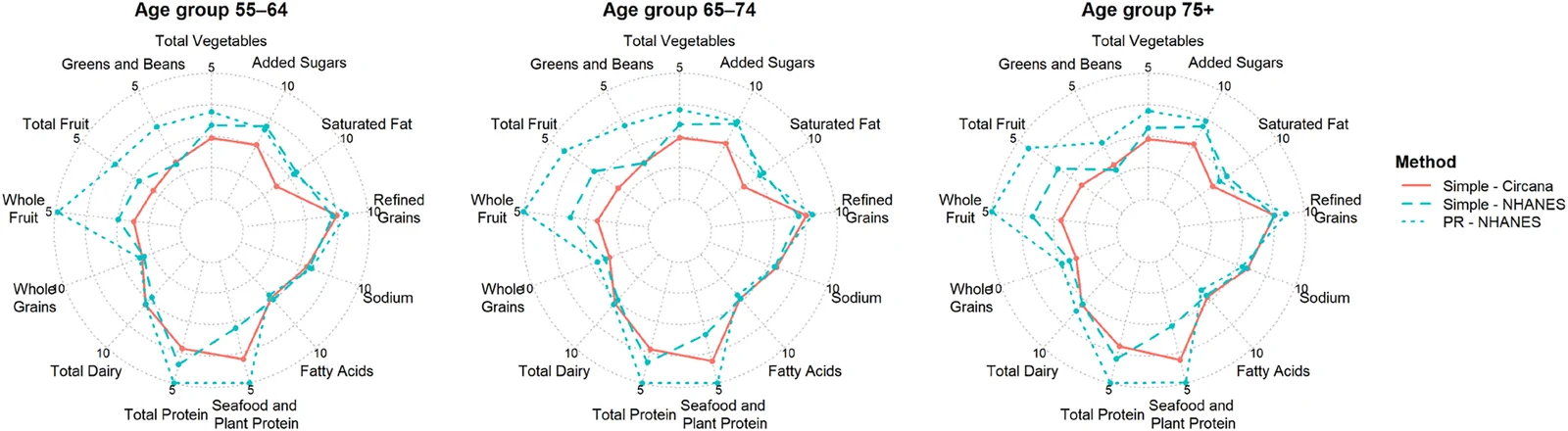
HEI component scores in older adult participants of NHANES and Circana (CN), using analysis method. HEI, Healthy Eating Index.

Figure 5 illustrates the comparison between the simple means and PR methods for calculating HEI component scores in NHANES compared with simple means alone in CN. In NHANES, the PR method, which estimates population-level habitual intake, produced higher scores for greens and beans, total fruit, whole fruit, and seafood and plant proteins—with whole fruit, seafood and plant protein, and total protein achieving perfect adherence levels. There were smaller or negligible differences in other components. No significant differences were found for refined grains, total protein foods, fatty acids, sodium, or added sugars. Simple means values in NHANES were more consistently aligned with simple means results in CN. For consistency with CN’s full-year household-level data, the simple means method was used as the primary approach in both datasets.

### Food group composition by mass and energy

**FIGURE 6:**
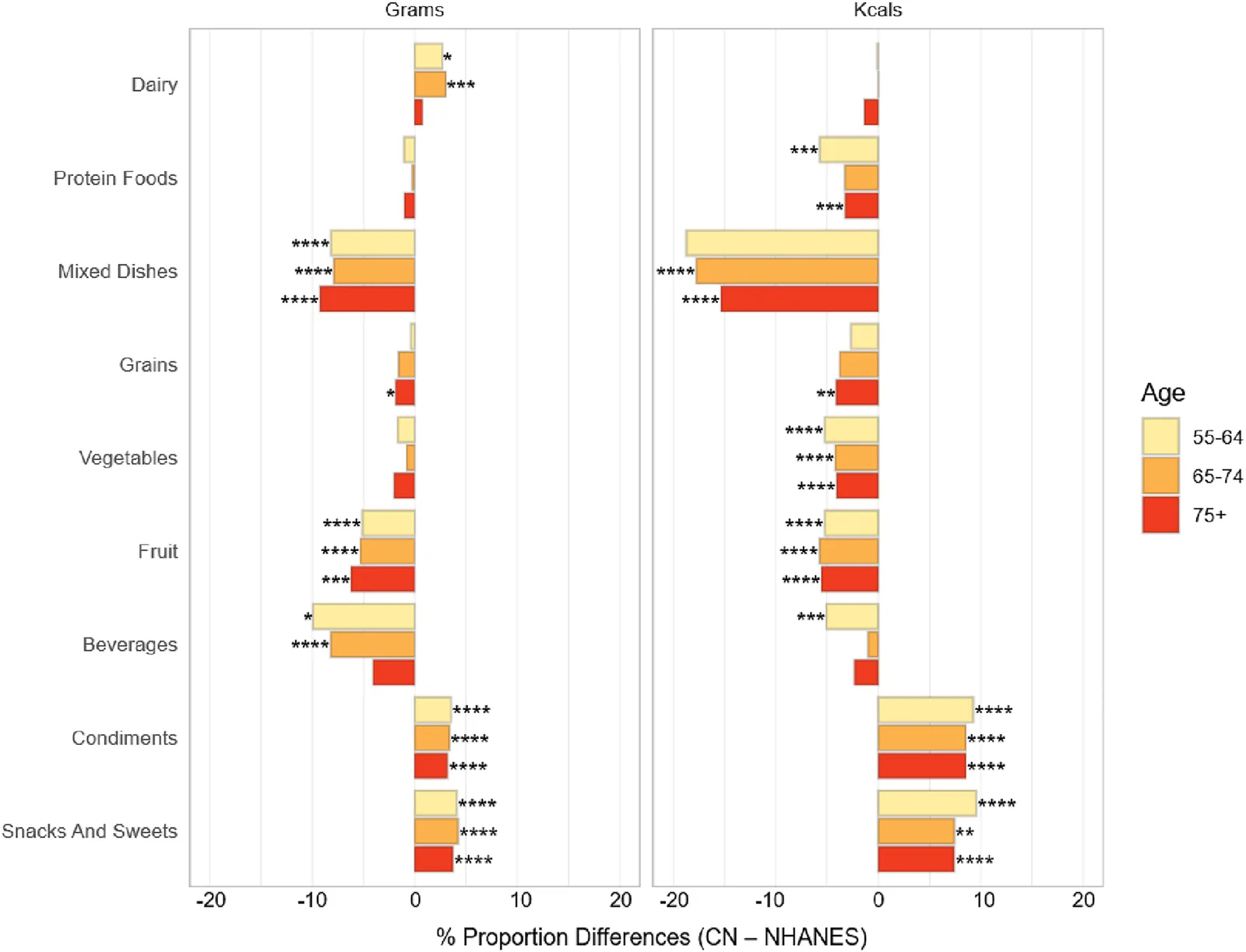
Proportions of DGAC major food groups in Circana (CN) relative to NHANES by mass (grams) and energy (kcals). Bars to the right indicate a higher proportion in CN. ∗ = <0.05, ∗∗ = <0.01, ∗∗∗ = <0.001, and ∗∗∗∗ = <0.0001. DGAC, Dietary Guidelines Advisory Committee.

Figure 6 displays differences in food group composition of CN with respect to NHANES, using DGAC major food group categories expressed as a percentage of total energy intake (kilocalories) and percentage by mass (grams). Bars to the right (positive difference) indicate a higher proportion in the CN data, whereas bars to the left (negative difference) indicate a lower proportion in CN compared with NHANES. Overall, the absolute differences in contribution by mass were <10% for all food groups, with particularly close alignment of 5% or less for dairy, protein foods, grains, vegetables, condiments, and snacks and sweets, across all age categories—although some qualified as significantly different. By caloric contribution, only mixed dishes showed >10% difference, at 15% to 18% in favor of NHANES. Condiments, snacks, and sweets were slightly shy of 10% in favor of CN. Only dairy and grains showed <5% difference in caloric contribution across all ages, but this list extended to include protein foods, vegetables, and beverages when excepting the youngest age group, who showed differences at or slightly above 5%.

Age-stratified analysis in NHANES revealed broader variability in food group composition, particularly in dairy, protein foods, grains, and beverages. In CN, variation by age was less pronounced. Differences by sex were more apparent in NHANES for beverages and mixed dishes, whereas in CN, snacks, condiments, and beverages showed more marked variation across male-only, female-only, and mixed-sex households (data not shown).

## Discussion

Our study compared dietary quality and food group proportions among grocery/store-bought foods for United States adults aged 55 y and older using 2 nationally representative but methodologically distinct datasets. For diet quality, we found that the mean total HEI scores of NHANES and CN were 55.7 and 51.3, respectively, which may be interpreted as a grade of F (i.e., “failing”) for dietary healthfulness [25]. The 4.4-point difference between the 2 datasets is below the 5- to 6-point threshold to be considered practically meaningful [25], indicating overall alignment. Within each dataset, median total HEI was only slightly higher in oldest compared with youngest age groups. This demonstrates that both datasets were consistent in showing that there is suboptimal adherence to dietary guidelines among older Americans, whether in individual consumption or in household purchase of grocery foods, consistent with previous reports [1,4].

Our analysis further demonstrated underlying variability in what the total HEI scores reflect. Comparisons of individual HEI component scores within the NHANES and CN datasets showed values from <30% to >80% of maximum adherence to individual component guidelines (out of 5 or 10 points), indicating a wide range in how dietary targets are being met. There was evidence of consistent shortfalls in both consumption and purchase of total fruit, whole fruit, greens and beans, and whole grains—components for which older adults regularly fall below dietary recommendations [4]. These generally showed improved scores with older age, with the exception of greens and beans (Supplemental Table 1). However, it should be noted that random-weight items have limited representation in CN because data do not capture purchase quantity [28], which disproportionately affects certain components such as total/whole fruit. Overall, our findings indicate that although older adults may adopt healthier food purchasing and dietary behaviors with age for certain food types, there are clear targets for further study and potential improvement.

The majority of HEI component scores showed 0% to 10% difference between the NHANES and CN datasets, indicating many points of alignment (Figure 4). The largest differences in HEI components scores (20% and above) between the 2 datasets were for seafood and plant proteins, saturated fat, added sugar, and total/whole fruit. These findings are similar to other studies using both NHANES and CN datasets [28], and the inconsistencies observed for some component scores may reflect differences in how food items are reported compared with purchased (e.g., for episodic rather than habitually consumed foods). Added sugars and saturated fats may be underestimated in self-report due to social desirability, as foods high in fat and/or carbohydrates are often linked to underreporting [29]. This would mean higher HEI component scores in NHANES, as the appearance would be toward better meeting moderation guidelines. Meanwhile, fish, shellfish, and legumes are considered episodically consumed foods in HEI analysis [22], leading to likely lower representation in the 2 dietary recalls than in year-long grocery purchase records, meaning lower scores for the seafood and plant proteins component in NHANES than in CN. Yet, there are also limitations in fully capturing food purchases, particularly of certain food types [15,29,30], providing further evidence that each data source includes distinct facets of dietary behavior.

For major food groups, the majority of their proportions were similar between consumption (NHANES) and purchase (CN), both by the percentage mass and percentage energy of total—despite dataset differences in relevant factors such as timespan in data collection (24-h dietary recalls for individuals in NHANES, total year-long food purchases by households in CN). However, certain food groups showed meaningful mismatches in the context of their direction of difference: mixed dishes, condiments (including spreads, gravies, and salad dressing), and snacks and sweets (Figure 6). NHANES showed a significantly higher proportion of mixed foods by both energy and mass, which can be due to its reporting of foods as consumed, whereas the CN would only capture these foods if they were bought in the final form, rather than their ingredients. Such multi-ingredient dishes typically include 2 or more distinct food groups combined as a single item, such as a meat and vegetable stew, pizza, stir-fry, etc. In this way, we reflect differences in data granularity (assembled dishes compared with ingredients), as NHANES provides valuable additional information on how the food is ultimately consumed out of the many possible combinations of the underlying ingredients. Fruit also had significantly higher representation in NHANES consumption than CN purchases, which is largely attributable to the difficulty in CN of capturing random-weight items [31], and warrants further examination for how this information may be obtained in purchase datasets for nonprepacked items to be more accurately represented. Meanwhile, the condiments, snacks, and sweets were lower in NHANES compared with CN. This discrepancy may be due to underreporting of discretionary foods (e.g., snack foods, sweetened beverages, and flavorful sauces) in dietary recalls, especially when consumed incidentally or between meals [8]. Receipt-based data are meant to record store-bought foods brought into the household, considered to be an objective measure less dependent on memory-based dietary reporting [[32], [33], [34]]. However, purchase data may be vulnerable to other factors, such as selective participation in long-term panels, the time-consuming nature of the recording process, and missing or incorrectly recorded grocery trip data [30].

Overall, these findings align with previous studies showing moderate correspondence between food purchase and dietary intake data [[13], [14], [15]]. However, in those studies, the individual food consumption and household food purchase data were obtained from within the same population—which is rarely available or possible in large-scale epidemiologic studies. Some degree of discrepancy in percent composition of certain food groups between CN compared with NHANES is expected. Indeed, this reinforces the premise that food purchase and consumption data each provide valuable but distinct windows into dietary behavior and complement each other by providing nonoverlapping information. Inconsistencies between intake and purchase point to areas of further study.

## Strengths and Limitations

This study has several notable strengths. To our knowledge, it is the first to compare dietary intake and household food purchase patterns specifically among United States older adults using 2 nationally representative datasets—NHANES and CN—linked by a common dietary quality framework (i.e., the HEI-2015). By restricting the analysis to grocery/store-bought food from the same time period and aligning scoring methods, we ensured meaningful comparability across sources. Stratifying analysis by age and sex allowed us to explore demographic variability in diet quality and food group composition, whereas the inclusion of both energy- and mass-based comparisons provided complementary views of dietary patterns.

This study also highlights the potential of using multiple data streams to enhance dietary surveillance, particularly for aging populations. NHANES offers rich individual-level dietary, clinical, and sociodemographic data, but is resource-intensive and constrained by recall limitations. CN provides real-time, high-volume data on long-term purchasing patterns, but our findings indicate that there remains variability—particularly for certain food categories.

The large sample size and methodological differences between dietary datasets can pose inherent challenges to direct equivalence. Although we attempted to address these by aligning sample structures and analytic approaches and prioritizing the magnitude and consistency of differences over statistical significance alone, still, several limitations remain. A summarized assessment of differences in the datasets that warrant consideration in analytical approaches include the following:

1) NHANES foods as eaten, CN as purchased: It should be considered that NHANES foods are reported as eaten, whereas CN reports the ingredients as individual grocery item purchases, and that there may be differences between what is purchased and what is actually consumed—due to food waste, sharing/uneven distribution within households, or use for purposes other than personal consumption [15].
2) NHANES as self-report: NHANES data are self-reported and subject to recall and social desirability biases, particularly among older adults who may experience cognitive or memory challenges. Although memory-based methods are criticized for being subjective and prone to energy underreporting and occasionally implausible food intake, these limitations have long been recognized and studied to develop augmented data collection methods and analytical adjustments to improve validity [8]. Countermeasures include repeated dietary measures using multiple instruments, use of trained interviewers for diet record collections, validation against biomarkers and biological samples, and statistical adjustment to reduce inflated variance and better estimate habitual intake, among others [7].
3) Restriction to grocery/store-bought foods in NHANES to match CN: CN data are collected at the household level and do not capture food consumed away from home, such as meals from restaurants or institutions. We restricted NHANES data to grocery/store-bought foods for comparability. In exploratory assessment, grocery/store-bought foods represented ∼70% of total NHANES dietary intake by source classification; therefore, results should be interpreted as comparisons of grocery/store-sourced intake and household grocery purchases, not total diet. Some studies have shown older adults in the United States have reduced consumption of commercially prepared meals outside the home and lower spending on eating out [35,36].
4) CN limitations in random-weight capture: Certain foods—such as random-weight produce and other items purchased by weight rather than by package, as well as prepared meals—may be underreported in scanner-based data, because purchased quantity is not recorded [18,28].
5) CN PPC limited to sufficient purchases (90% of sales): Although the PPC enhances the nutritional characterization of purchases, it is limited to UPCs with sufficient purchases (although still ∼90% of sales) [17].
6) NHANES 2-d dietary recall is not habitual: Simple means method was used to calculate HEI in NHANES for comparability to CN, which reflects household-level purchasing over time; PR calculations produced small shifts that did not change conclusions. Nevertheless, it is important to note that the distributions of HEI scores in NHANES may appear more variable than other methods that adjust for within-person variability.
7) Dataset units of individual compared with household level: Differences in unit of analysis—individuals in NHANES compared with households in CN—limit direct comparability, although restricting the sample to 1- and 2-person households helps mitigate this issue. However, we note that limiting CN to 1 to 2 person households improves comparability but may reduce generalizability to larger/younger households.
8) CN and NHANES sample comparison: Different sampling approaches were used for the 2 datasets, although both were weighted for population representation at the national level. Significant differences were found only in some categories of marital status and education level across the datasets (Table 2). Nevertheless, some skewness in the socioeconomic condition of CN participants, as well as factors influencing consistent reporting of purchasing in CN, may impact generalizability and representation of more vulnerable/underrepresented population groups [18]. The datasets were not combined, and so individual-level comparisons could not be made; rather, group means were compared at the population group level for age and/or sex.

With the above factors in mind, despite many points of alignment in CN data, household purchase data, and NHANES dietary intake data, inconsistencies remain. Indeed, these highlight the need for further studies to quantify the error terms between consumption and purchase. In the meantime, leveraging both sources, aligned through consistent scoring frameworks, may offer a more complete picture of dietary behavior. These data streams may inform precision nutrition strategies when linked longitudinally to individual health and biological outcomes—especially with the inclusion of data on household and neighborhood characteristics and the retail food environment.

Finally, the current study was limited to a cross-sectional design, but future longitudinal work could test whether purchase patterns predict sustained improvements (or declines) in diet quality and health outcomes. Linking these datasets over time and connecting them to social and health indicators would allow for more precise estimation of how food environments and purchasing power shape nutritional trajectories in aging populations.

In conclusion, this study of adults aged ≥55 y, grocery/store-sourced dietary intake from NHANES and household grocery purchases from Circana showed moderate population-level alignment in total HEI scores and several food group patterns, whereas diverging for specific categories such as fruit, seafood and plant proteins, mixed dishes, condiments, and snacks/sweets. Despite methodological differences, both data sources consistently indicated that grocery/store-sourced dietary intake and household food purchases suggest persistent shortfalls in several dietary components relevant to guideline adherence, particularly fruit, vegetables, whole grains, and saturated fat moderation. These findings should not be interpreted as evidence of individual-level validity or direct equivalence between purchase and intake data. Rather, they show that recall-based and purchase-based data capture distinct dimensions of dietary behavior.

The integration of NHANES and CN data demonstrates the complementary strengths of self-reported and behavioral data streams in capturing the dietary landscape of aging populations. Whereas NHANES provides individual-level intake and health context, CN delivers longitudinal household acquisition patterns, product-level detail, and potential linkage to price and retail environments. Taken together, these sources may help identify both behavioral targets and structural barriers to healthy eating in later life.

In a precision nutrition framework, purchase data may help move dietary surveillance from episodic self-report toward longitudinal behavioral phenotyping, capturing the foods that enter the household, their cost, frequency, retail context, and potential relationship to neighborhood food environments and health trajectories. Together, these findings highlight the potential of harmonizing recall- and purchase-based metrics to enhance dietary surveillance, guide policy, and support precision nutrition research for healthy aging.

## Author contributions

The authors’ responsibilities were as follows – NHE-A, JMO, ACC, JR, ML, EM: contributed to investigation and conceptualization; ML, JR, FN, NHE-A, CMF: contributed to data curation and formal analysis; NHE-A, JMO, CMF, JR, ML: contributed to writing; ACC, MKS, EM, LDP, C-QL, JMO: contributed to review and editing; and all authors: meaningfully contributed to the manuscript, with the above designation of primary roles.

## Data availability

NHANES is a public USDA database. The Circana Consumer Network is a proprietary database; access is through an application process. The findings of this study should not be attributed to Circana.

## Declaration of Generative AI and AI-assisted technologies in the writing process

The authors declare that no generative AI or AI-assisted technologies were used in the writing of this manuscript.

## Funding

This research was supported in part by the USDA, Agricultural Research Service, under Cooperative Agreement no. 58-8050-3-003. The funders had no role in the design of the study; in the collection, analysis, or interpretation of data; in the writing of the manuscript; or in the decision to publish the results. This research was also supported in part by the USDA, Economic Research Service. The findings and conclusions in this publication are those of the authors and should not be construed to represent any official USDA or United States Government determination or policy.

## Conflict of interest

The authors report no conflicts of interest. MKS is an Editorial Board Member/Editor for *Current Developments in Nutrition* and played no role in the Journal's evaluation of the manuscript
