# Supplementary material for: Aligning Dietary Intake and Food Purchase Data in United States Older Adults: Insights from the National Health and Nutrition Examination Survey and Circana Consumer Network
Source: Curr Dev Nutr. 2026 Jun 29;10(8):109421. doi: 10.1016/j.cdnut.2026.109421 (PMC13524882; doi:10.1016/j.cdnut.2026.109421)
Supplement: Supplementary file 1 [file mmc1.docx]

**Supplemental Table 1:** HEI total and component mean scores and variance for Circana (CN) and NHANES, among all and by population subgroup (age and sex), with t-statistic and adjusted p-values from comparison tests.

| Sex | Age group | HEI component | CN mean | | NHANES mean | CN var | NHANES var | t-statistic | p-value | Adjusted  p-value |
| --- | --- | --- | --- | --- | --- | --- | --- | --- | --- | --- |
| all | all | Total Vegetables | 2.42 | 2.93 | | 0.0000 | 0.0086 | -5.42 | 7.11E-05 | <0.0001 |
| Female | all | Total Vegetables | 2.36 | 3.17 | | 0.0002 | 0.0104 | -7.89 | 1.03E-06 | <0.0001 |
| Male | all | Total Vegetables | 2.28 | 2.63 | | 0.0007 | 0.0119 | -3.11 | 0.00714 | 0.20002 |
| all | 55-64 | Total Vegetables | 2.43 | 2.94 | | 0.0001 | 0.0229 | -3.37 | 0.00424 | 0.17802 |
| all | 65-74 | Total Vegetables | 2.45 | 2.97 | | 0.0001 | 0.0136 | -4.51 | 4.18E-04 | 0.01756 |
| all | 75+ | Total Vegetables | 2.39 | 2.81 | | 0.0002 | 0.0285 | -2.51 | 0.02418 | 1 |
| Female | 55-64 | Total Vegetables | 2.36 | 3.26 | | 0.0005 | 0.0299 | -5.19 | 1.10E-04 | 0.00928 |
| Female | 65-74 | Total Vegetables | 2.39 | 3.09 | | 0.0005 | 0.0221 | -4.66 | 3.10E-04 | 0.02605 |
| Female | 75+ | Total Vegetables | 2.34 | 3.08 | | 0.0009 | 0.0326 | -4.05 | 0.00104 | 0.08771 |
| Male | 55-64 | Total Vegetables | 2.23 | 2.55 | | 0.0014 | 0.0325 | -1.73 | 0.10343 | 1 |
| Male | 65-74 | Total Vegetables | 2.36 | 2.84 | | 0.0016 | 0.0157 | -3.66 | 0.00231 | 0.19416 |
| Male | 75+ | Total Vegetables | 2.26 | 2.46 | | 0.0038 | 0.0353 | -1.02 | 0.32397 | 1 |
| all | all | Greens and Beans | 1.78 | 1.70 | | 0.0001 | 0.0070 | 0.90 | 0.3843 | 1 |
| Female | all | Greens and Beans | 1.81 | 1.89 | | 0.0003 | 0.0126 | -0.71 | 0.48635 | 1 |
| Male | all | Greens and Beans | 1.53 | 1.47 | | 0.0009 | 0.0106 | 0.51 | 0.61683 | 1 |
| all | 55-64 | Greens and Beans | 1.81 | 1.73 | | 0.0001 | 0.0146 | 0.68 | 0.50843 | 1 |
| all | 65-74 | Greens and Beans | 1.79 | 1.77 | | 0.0001 | 0.0103 | 0.19 | 0.85026 | 1 |
| all | 75+ | Greens and Beans | 1.69 | 1.48 | | 0.0003 | 0.0105 | 2.04 | 0.05991 | 1 |
| Female | 55-64 | Greens and Beans | 1.85 | 2.07 | | 0.0008 | 0.0257 | -1.32 | 0.20562 | 1 |
| Female | 65-74 | Greens and Beans | 1.82 | 1.85 | | 0.0007 | 0.0218 | -0.17 | 0.86921 | 1 |
| Female | 75+ | Greens and Beans | 1.75 | 1.49 | | 0.0013 | 0.0176 | 1.86 | 0.08285 | 1 |
| Male | 55-64 | Greens and Beans | 1.47 | 1.33 | | 0.0018 | 0.0157 | 1.06 | 0.30479 | 1 |
| Male | 65-74 | Greens and Beans | 1.62 | 1.69 | | 0.0022 | 0.0210 | -0.48 | 0.6352 | 1 |
| Male | 75+ | Greens and Beans | 1.50 | 1.46 | | 0.0046 | 0.0444 | 0.15 | 0.8839 | 1 |
| all | all | Total Fruit | 1.72 | 2.61 | | 0.0001 | 0.0112 | -8.37 | 4.93E-07 | <0.0001 |
| Female | all | Total Fruit | 1.70 | 2.72 | | 0.0003 | 0.0129 | -8.91 | 2.22E-07 | <0.0001 |
| Male | all | Total Fruit | 1.79 | 2.47 | | 0.0010 | 0.0162 | -5.19 | 1.10E-04 | 0.00308 |
| all | 55-64 | Total Fruit | 1.56 | 2.24 | | 0.0001 | 0.0193 | -4.89 | 1.96E-04 | 0.00825 |
| all | 65-74 | Total Fruit | 1.72 | 2.90 | | 0.0001 | 0.0136 | -10.07 | 4.54E-08 | <0.0001 |
| all | 75+ | Total Fruit | 1.95 | 3.09 | | 0.0003 | 0.0278 | -6.78 | 6.25E-06 | <0.0001 |
| Female | 55-64 | Total Fruit | 1.55 | 2.45 | | 0.0006 | 0.0347 | -4.81 | 2.31E-04 | 0.01939 |
| Female | 65-74 | Total Fruit | 1.68 | 2.91 | | 0.0006 | 0.0176 | -9.12 | 1.66E-07 | <0.0001 |
| Female | 75+ | Total Fruit | 1.86 | 3.10 | | 0.0011 | 0.0217 | -8.23 | 6.08E-07 | <0.0001 |
| Male | 55-64 | Total Fruit | 1.60 | 1.99 | | 0.0017 | 0.0191 | -2.70 | 0.01648 | 1 |
| Male | 65-74 | Total Fruit | 1.76 | 2.88 | | 0.0024 | 0.0377 | -5.59 | 5.14E-05 | 0.00432 |
| Male | 75+ | Total Fruit | 2.10 | 3.07 | | 0.0062 | 0.0579 | -3.87 | 0.00153 | 0.12815 |
| all | all | Whole Fruit | 2.02 | 2.84 | | 0.0001 | 0.0162 | -6.47 | 1.06E-05 | <0.0001 |
| Female | all | Whole Fruit | 2.05 | 2.99 | | 0.0004 | 0.0239 | -6.04 | 2.25E-05 | <0.0001 |
| Male | all | Whole Fruit | 1.81 | 2.66 | | 0.0011 | 0.0198 | -5.93 | 2.74E-05 | <0.0001 |
| all | 55-64 | Whole Fruit | 1.85 | 2.47 | | 0.0001 | 0.0328 | -3.41 | 0.00384 | 0.16130 |
| all | 65-74 | Whole Fruit | 2.04 | 3.12 | | 0.0002 | 0.0174 | -8.13 | 7.03E-07 | <0.0001 |
| all | 75+ | Whole Fruit | 2.23 | 3.37 | | 0.0004 | 0.0229 | -7.47 | 1.97E-06 | <0.0001 |
| Female | 55-64 | Whole Fruit | 1.85 | 2.74 | | 0.0008 | 0.0596 | -3.65 | 0.00236 | 0.19842 |
| Female | 65-74 | Whole Fruit | 2.07 | 3.16 | | 0.0008 | 0.0120 | -9.68 | 7.69E-08 | <0.0001 |
| Female | 75+ | Whole Fruit | 2.24 | 3.36 | | 0.0016 | 0.0154 | -8.65 | 3.27E-07 | <0.0001 |
| Male | 55-64 | Whole Fruit | 1.64 | 2.15 | | 0.0021 | 0.0486 | -2.25 | 0.03976 | 1 |
| Male | 65-74 | Whole Fruit | 1.83 | 3.07 | | 0.0027 | 0.0484 | -5.52 | 5.92E-05 | 0.00497 |
| Male | 75+ | Whole Fruit | 2.02 | 3.37 | | 0.0061 | 0.0494 | -5.76 | 3.78E-05 | 0.00317 |
| all | all | Whole Grains | 3.48 | 3.60 | | 0.0002 | 0.0531 | -0.53 | 0.6015 | 1 |
| Female | all | Whole Grains | 3.41 | 3.75 | | 0.0010 | 0.0699 | -1.26 | 0.22646 | 1 |
| Male | all | Whole Grains | 3.66 | 3.43 | | 0.0037 | 0.0644 | 0.89 | 0.38664 | 1 |
| all | 55-64 | Whole Grains | 3.38 | 3.24 | | 0.0004 | 0.0935 | 0.45 | 0.66218 | 1 |
| all | 65-74 | Whole Grains | 3.48 | 3.83 | | 0.0004 | 0.1178 | -1.00 | 0.33318 | 1 |
| all | 75+ | Whole Grains | 3.62 | 4.19 | | 0.0011 | 0.0433 | -2.69 | 0.01694 | 0.71162 |
| Female | 55-64 | Whole Grains | 3.31 | 3.53 | | 0.0020 | 0.0865 | -0.73 | 0.47888 | 1 |
| Female | 65-74 | Whole Grains | 3.40 | 3.86 | | 0.0022 | 0.2655 | -0.88 | 0.39059 | 1 |
| Female | 75+ | Whole Grains | 3.52 | 4.14 | | 0.0045 | 0.0965 | -1.94 | 0.07195 | 1 |
| Male | 55-64 | Whole Grains | 3.58 | 2.91 | | 0.0078 | 0.1403 | 1.74 | 0.10203 | 1 |
| Male | 65-74 | Whole Grains | 3.61 | 3.79 | | 0.0081 | 0.1155 | -0.50 | 0.62479 | 1 |
| Male | 75+ | Whole Grains | 3.84 | 4.25 | | 0.0208 | 0.1717 | -0.95 | 0.35917 | 1 |
| all | all | Total Dairy | 5.37 | 4.85 | | 0.0002 | 0.0222 | 3.47 | 0.00342 | 0.04787 |
| Female | all | Total Dairy | 5.20 | 4.99 | | 0.0010 | 0.0383 | 1.05 | 0.31143 | 1 |
| Male | all | Total Dairy | 5.33 | 4.68 | | 0.0033 | 0.0303 | 3.53 | 0.00303 | 0.08482 |
| all | 55-64 | Total Dairy | 5.42 | 4.63 | | 0.0004 | 0.0487 | 3.55 | 0.00291 | 0.12239 |
| all | 65-74 | Total Dairy | 5.27 | 4.90 | | 0.0004 | 0.0471 | 1.70 | 0.1089 | 1 |
| all | 75+ | Total Dairy | 5.42 | 5.35 | | 0.0011 | 0.0301 | 0.43 | 0.67559 | 1 |
| Female | 55-64 | Total Dairy | 5.24 | 4.82 | | 0.0022 | 0.0729 | 1.52 | 0.14881 | 1 |
| Female | 65-74 | Total Dairy | 5.09 | 4.97 | | 0.0023 | 0.1042 | 0.38 | 0.71271 | 1 |
| Female | 75+ | Total Dairy | 5.26 | 5.45 | | 0.0044 | 0.0490 | -0.82 | 0.42381 | 1 |
| Male | 55-64 | Total Dairy | 5.35 | 4.41 | | 0.0070 | 0.1050 | 2.82 | 0.013 | 1 |
| Male | 65-74 | Total Dairy | 5.13 | 4.83 | | 0.0081 | 0.0348 | 1.45 | 0.16816 | 1 |
| Male | 75+ | Total Dairy | 5.55 | 5.21 | | 0.0179 | 0.0785 | 1.10 | 0.28718 | 1 |
| all | all | Total Protein | 3.58 | 4.17 | | 0.0000 | 0.0045 | -8.75 | 2.81E-07 | <0.0001 |
| Female | all | Total Protein | 3.47 | 4.19 | | 0.0002 | 0.0090 | -7.47 | 1.99E-06 | <0.0001 |
| Male | all | Total Protein | 3.65 | 4.15 | | 0.0007 | 0.0086 | -5.20 | 1.07E-04 | 0.00301 |
| all | 55-64 | Total Protein | 3.59 | 4.24 | | 0.0001 | 0.0084 | -7.08 | 3.74E-06 | <0.0001 |
| all | 65-74 | Total Protein | 3.62 | 4.14 | | 0.0001 | 0.0170 | -3.98 | 0.00121 | 0.05101 |
| all | 75+ | Total Protein | 3.49 | 4.02 | | 0.0002 | 0.0305 | -2.98 | 0.00931 | 0.39120 |
| Female | 55-64 | Total Protein | 3.45 | 4.25 | | 0.0005 | 0.0174 | -5.96 | 2.63E-05 | 0.00221 |
| Female | 65-74 | Total Protein | 3.53 | 4.17 | | 0.0005 | 0.0289 | -3.77 | 0.00183 | 0.15413 |
| Female | 75+ | Total Protein | 3.44 | 4.06 | | 0.0010 | 0.0238 | -3.91 | 0.00138 | 0.11580 |
| Male | 55-64 | Total Protein | 3.60 | 4.23 | | 0.0015 | 0.0085 | -6.33 | 1.35E-05 | 0.00114 |
| Male | 65-74 | Total Protein | 3.76 | 4.11 | | 0.0015 | 0.0233 | -2.23 | 0.04141 | 1 |
| Male | 75+ | Total Protein | 3.57 | 3.96 | | 0.0034 | 0.0561 | -1.60 | 0.13121 | 1 |
| all | all | Seafood & Plant Protein | 4.06 | 2.82 | | 0.0001 | 0.0110 | 11.76 | 5.72E-09 | <0.0001 |
| Female | all | Seafood & Plant Protein | 3.93 | 2.96 | | 0.0003 | 0.0189 | 6.98 | 4.42E-06 | <0.0001 |
| Male | all | Seafood & Plant Protein | 3.86 | 2.66 | | 0.0009 | 0.0173 | 8.85 | 2.43E-07 | <0.0001 |
| all | 55-64 | Seafood & Plant Protein | 4.02 | 2.76 | | 0.0001 | 0.0229 | 8.35 | 5.08E-07 | <0.0001 |
| all | 65-74 | Seafood & Plant Protein | 4.10 | 3.02 | | 0.0001 | 0.0371 | 5.61 | 4.97E-05 | 0.00209 |
| all | 75+ | Seafood & Plant Protein | 4.06 | 2.67 | | 0.0003 | 0.0341 | 7.48 | 1.95E-06 | <0.0001 |
| Female | 55-64 | Seafood & Plant Protein | 3.86 | 2.99 | | 0.0007 | 0.0271 | 5.22 | 1.03E-04 | 0.00865 |
| Female | 65-74 | Seafood & Plant Protein | 3.94 | 2.90 | | 0.0007 | 0.0568 | 4.35 | 5.75E-04 | 0.04834 |
| Female | 75+ | Seafood & Plant Protein | 3.97 | 2.98 | | 0.0012 | 0.0380 | 5.03 | 1.50E-04 | 0.01264 |
| Male | 55-64 | Seafood & Plant Protein | 3.75 | 2.48 | | 0.0020 | 0.0472 | 5.76 | 3.79E-05 | 0.00319 |
| Male | 65-74 | Seafood & Plant Protein | 3.92 | 3.15 | | 0.0023 | 0.0428 | 3.61 | 0.00257 | 0.21613 |
| Male | 75+ | Seafood & Plant Protein | 3.93 | 2.26 | | 0.0050 | 0.0660 | 6.26 | 1.53E-05 | 0.00128 |
| all | all | Fatty Acids | 4.66 | 4.76 | | 0.0003 | 0.0397 | -0.49 | 0.62925 | 1 |
| Female | all | Fatty Acids | 4.53 | 4.65 | | 0.0016 | 0.0735 | -0.46 | 0.65276 | 1 |
| Male | all | Fatty Acids | 4.98 | 4.89 | | 0.0046 | 0.0291 | 0.49 | 0.62806 | 1 |
| all | 55-64 | Fatty Acids | 4.66 | 4.88 | | 0.0006 | 0.0514 | -0.96 | 0.35094 | 1 |
| all | 65-74 | Fatty Acids | 4.74 | 4.77 | | 0.0007 | 0.1412 | -0.09 | 0.92988 | 1 |
| all | 75+ | Fatty Acids | 4.58 | 4.44 | | 0.0017 | 0.0585 | 0.57 | 0.57983 | 1 |
| Female | 55-64 | Fatty Acids | 4.55 | 4.75 | | 0.0036 | 0.0822 | -0.67 | 0.5141 | 1 |
| Female | 65-74 | Fatty Acids | 4.62 | 4.49 | | 0.0037 | 0.2451 | 0.27 | 0.79024 | 1 |
| Female | 75+ | Fatty Acids | 4.41 | 4.68 | | 0.0070 | 0.0882 | -0.88 | 0.39449 | 1 |
| Male | 55-64 | Fatty Acids | 5.09 | 5.03 | | 0.0097 | 0.0960 | 0.18 | 0.86277 | 1 |
| Male | 65-74 | Fatty Acids | 5.13 | 5.09 | | 0.0111 | 0.1204 | 0.11 | 0.91712 | 1 |
| Male | 75+ | Fatty Acids | 4.65 | 4.11 | | 0.0248 | 0.1607 | 1.27 | 0.2244 | 1 |
| all | all | Sodium | 5.71 | 5.72 | | 0.0004 | 0.0240 | -0.10 | 0.91918 | 1 |
| Female | all | Sodium | 5.80 | 5.79 | | 0.0021 | 0.0396 | 0.01 | 0.99079 | 1 |
| Male | all | Sodium | 5.89 | 5.64 | | 0.0058 | 0.0645 | 0.93 | 0.36875 | 1 |
| all | 55-64 | Sodium | 5.56 | 5.79 | | 0.0008 | 0.0488 | -1.03 | 0.31918 | 1 |
| all | 65-74 | Sodium | 5.71 | 5.56 | | 0.0009 | 0.0691 | 0.54 | 0.59573 | 1 |
| all | 75+ | Sodium | 5.93 | 5.83 | | 0.0023 | 0.0532 | 0.40 | 0.69427 | 1 |
| Female | 55-64 | Sodium | 5.60 | 6.00 | | 0.0050 | 0.0991 | -1.22 | 0.24109 | 1 |
| Female | 65-74 | Sodium | 5.80 | 5.35 | | 0.0048 | 0.1031 | 1.36 | 0.19397 | 1 |
| Female | 75+ | Sodium | 5.98 | 5.99 | | 0.0093 | 0.0964 | -0.02 | 0.98284 | 1 |
| Male | 55-64 | Sodium | 5.68 | 5.54 | | 0.0118 | 0.1380 | 0.36 | 0.72325 | 1 |
| Male | 65-74 | Sodium | 5.96 | 5.81 | | 0.0135 | 0.1297 | 0.41 | 0.6848 | 1 |
| Male | 75+ | Sodium | 6.10 | 5.63 | | 0.0324 | 0.0834 | 1.38 | 0.18929 | 1 |
| all | all | Refined Grains | 7.58 | 7.20 | | 0.0002 | 0.0268 | 2.27 | 0.03823 | 0.53516 |
| Female | all | Refined Grains | 7.63 | 7.21 | | 0.0010 | 0.0245 | 2.64 | 0.01861 | 0.52114 |
| Male | all | Refined Grains | 7.77 | 7.20 | | 0.0029 | 0.0565 | 2.36 | 0.03197 | 0.89520 |
| all | 55-64 | Refined Grains | 7.53 | 7.19 | | 0.0004 | 0.0750 | 1.24 | 0.23381 | 1 |
| all | 65-74 | Refined Grains | 7.61 | 7.01 | | 0.0004 | 0.0385 | 3.01 | 0.00885 | 0.37185 |
| all | 75+ | Refined Grains | 7.60 | 7.57 | | 0.0010 | 0.1142 | 0.10 | 0.92549 | 1 |
| Female | 55-64 | Refined Grains | 7.64 | 7.17 | | 0.0022 | 0.0958 | 1.49 | 0.15752 | 1 |
| Female | 65-74 | Refined Grains | 7.67 | 6.89 | | 0.0022 | 0.0554 | 3.24 | 0.00546 | 0.45889 |
| Female | 75+ | Refined Grains | 7.59 | 7.83 | | 0.0042 | 0.1467 | -0.62 | 0.54773 | 1 |
| Male | 55-64 | Refined Grains | 7.47 | 7.22 | | 0.0068 | 0.1393 | 0.67 | 0.51428 | 1 |
| Male | 65-74 | Refined Grains | 7.86 | 7.15 | | 0.0074 | 0.1077 | 2.10 | 0.05338 | 1 |
| Male | 75+ | Refined Grains | 8.10 | 7.23 | | 0.0130 | 0.1446 | 2.19 | 0.0446 | 1 |
| all | all | Saturated Fat | 3.70 | 5.53 | | 0.0003 | 0.0351 | -9.73 | 7.19E-08 | <0.0001 |
| Female | all | Saturated Fat | 3.60 | 5.54 | | 0.0014 | 0.0448 | -9.03 | 1.88E-07 | <0.0001 |
| Male | all | Saturated Fat | 4.50 | 5.52 | | 0.0045 | 0.0437 | -4.68 | 2.95E-04 | 0.00827 |
| all | 55-64 | Saturated Fat | 3.75 | 5.68 | | 0.0006 | 0.0515 | -8.45 | 4.34E-07 | <0.0001 |
| all | 65-74 | Saturated Fat | 3.65 | 5.57 | | 0.0006 | 0.0653 | -7.48 | 1.95E-06 | <0.0001 |
| all | 75+ | Saturated Fat | 3.72 | 5.09 | | 0.0015 | 0.0857 | -4.64 | 3.22E-04 | 0.01353 |
| Female | 55-64 | Saturated Fat | 3.69 | 5.56 | | 0.0033 | 0.0694 | -6.92 | 4.93E-06 | <0.0001 |
| Female | 65-74 | Saturated Fat | 3.56 | 5.70 | | 0.0033 | 0.1302 | -5.87 | 3.09E-05 | 0.00259 |
| Female | 75+ | Saturated Fat | 3.55 | 5.24 | | 0.0058 | 0.0886 | -5.50 | 6.08E-05 | 0.00511 |
| Male | 55-64 | Saturated Fat | 4.62 | 5.81 | | 0.0092 | 0.1217 | -3.31 | 0.00474 | 0.39780 |
| Male | 65-74 | Saturated Fat | 4.51 | 5.42 | | 0.0111 | 0.0844 | -2.94 | 0.01023 | 0.85915 |
| Male | 75+ | Saturated Fat | 4.30 | 4.88 | | 0.0252 | 0.1800 | -1.27 | 0.22361 | 1 |
| all | all | Added Sugars | 5.27 | 6.93 | | 0.0003 | 0.0186 | -12.13 | 3.73E-09 | <0.0001 |
| Female | all | Added Sugars | 4.92 | 6.95 | | 0.0014 | 0.0299 | -11.49 | 7.79E-09 | <0.0001 |
| Male | all | Added Sugars | 5.59 | 6.91 | | 0.0042 | 0.0211 | -8.32 | 5.29E-07 | <0.0001 |
| all | 55-64 | Added Sugars | 5.19 | 6.87 | | 0.0006 | 0.0716 | -6.27 | 1.50E-05 | <0.0001 |
| all | 65-74 | Added Sugars | 5.34 | 7.07 | | 0.0006 | 0.0343 | -9.26 | 1.35E-07 | <0.0001 |
| all | 75+ | Added Sugars | 5.28 | 6.85 | | 0.0014 | 0.0704 | -5.88 | 3.05E-05 | 0.00128 |
| Female | 55-64 | Added Sugars | 4.85 | 7.01 | | 0.0034 | 0.1120 | -6.35 | 1.31E-05 | 0.00110 |
| Female | 65-74 | Added Sugars | 5.00 | 7.07 | | 0.0034 | 0.0453 | -9.40 | 1.12E-07 | <0.0001 |
| Female | 75+ | Added Sugars | 4.90 | 6.61 | | 0.0059 | 0.0939 | -5.40 | 7.37E-05 | 0.00619 |
| Male | 55-64 | Added Sugars | 5.55 | 6.71 | | 0.0097 | 0.0682 | -4.16 | 8.32E-04 | 0.06990 |
| Male | 65-74 | Added Sugars | 5.63 | 7.07 | | 0.0104 | 0.0824 | -4.74 | 2.63E-04 | 0.0221 |
| Male | 75+ | Added Sugars | 5.59 | 7.18 | | 0.0207 | 0.1250 | -4.16 | 8.41E-04 | 0.07063 |
| all | all | Total Score | 51.34 | 55.68 | | 0.0037 | 0.8644 | -4.66 | 3.10E-04 | 0.00435 |
| Female | all | Total Score | 50.40 | 56.81 | | 0.0208 | 1.2226 | -5.75 | 3.85E-05 | 0.00108 |
| Male | all | Total Score | 52.63 | 54.32 | | 0.0565 | 0.9742 | -1.67 | 0.11536 | 1 |
| all | 55-64 | Total Score | 50.75 | 54.66 | | 0.0076 | 2.2416 | -2.61 | 0.01977 | 0.83052 |
| all | 65-74 | Total Score | 51.52 | 56.64 | | 0.0085 | 1.1753 | -4.71 | 2.79E-04 | 0.01174 |
| all | 75+ | Total Score | 51.96 | 56.76 | | 0.0223 | 1.2356 | -4.27 | 6.66E-04 | 0.02796 |
| Female | 55-64 | Total Score | 49.80 | 56.59 | | 0.0451 | 2.8545 | -3.99 | 0.00119 | 0.09992 |
| Female | 65-74 | Total Score | 50.58 | 56.42 | | 0.0477 | 1.2655 | -5.10 | 1.30E-04 | 0.01093 |
| Female | 75+ | Total Score | 50.81 | 58.01 | | 0.0920 | 1.6954 | -5.38 | 7.63E-05 | 0.00641 |
| Male | 55-64 | Total Score | 51.64 | 52.37 | | 0.1113 | 3.0426 | -0.41 | 0.68791 | 1 |
| Male | 65-74 | Total Score | 53.07 | 56.89 | | 0.1381 | 2.3856 | -2.41 | 0.02929 | 1 |
| Male | 75+ | Total Score | 53.50 | 55.08 | | 0.3191 | 2.3688 | -0.96 | 0.35171 | 1 |
