# Supplementary material for: Aligning Dietary Intake and Food Purchase Data in United States Older Adults: Insights from the National Health and Nutrition Examination Survey and Circana Consumer Network
Source: Curr Dev Nutr. 2026 Jun 29;10(8):109421. doi: 10.1016/j.cdnut.2026.109421 (PMC13524882; doi:10.1016/j.cdnut.2026.109421)
Supplement: Supplementary file 2 [file mmc2.docx]

**Supplementary Figure 1**: Circana (CN) sample household count by composition: 1- or 2-person Female (F, FF), 1- or 2-person Male (M, MM), and 2-person Male and Female (MF).
